# Room Temperature Exciton–Polariton Condensation in Silicon Metasurfaces Emerging from Bound States in the Continuum

**DOI:** 10.1021/acs.nanolett.3c01102

**Published:** 2023-06-13

**Authors:** Anton Matthijs Berghuis, Gabriel W. Castellanos, Shunsuke Murai, Jose Luis Pura, Diego R. Abujetas, Erik van Heijst, Mohammad Ramezani, José A. Sánchez-Gil, Jaime Gómez Rivas

**Affiliations:** † Department of Applied Physics and Science Education and Eindhoven Hendrik Casimir Institute, Eindhoven University of Technology, P.O. Box 513, 5600 MB Eindhoven, The Netherlands; ‡ Institute for Complex Molecular Systems-ICMS, Eindhoven University of Technology, P.O. Box 513, 5612 AJ, Eindhoven, The Netherlands; ¶ Department of Material Chemistry, Graduate School of Engineering, Kyoto University, Katsura, Nishikyo 6158510, Kyoto, Japan; ⊥ GdS-Optronlab, Física de la Materia Condensada, Universidad de Valladolid, Paseo de Belén 19, 47011 Valladolid, Spain; § Instituto de Estructura de la Materia (IEM-CSIC), Consejo Superior de Investigaciones Científicas, Serrano 121, 28006 Madrid, Spain; ∥ Physics Department, Fribourg University, Chemin de Musée 3, Fribourg 1700, Switzerland

**Keywords:** Bound State in the Continuum, Exciton−Polariton Condensation, Polariton Lasing, Strong Light−Matter Coupling, Metasurface, Surface Lattice Resonance

## Abstract

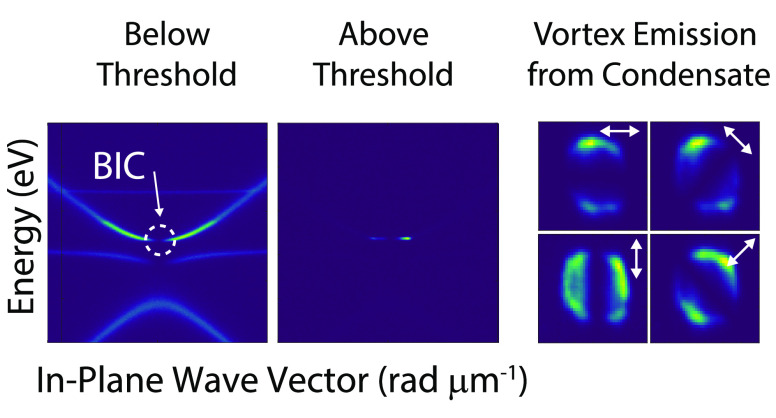

We show the first experimental demonstration of room-temperature exciton–polariton (EP) condensation from a bound state in the continuum (BIC). This demonstration is achieved by strongly coupling stable excitons in an organic perylene dye with the extremely long-lived BIC in a dielectric metasurface of silicon nanoparticles. The long lifetime of the BIC, mainly due to the suppression of radiation leakage, allows for EP thermalization to the ground state before decaying. This property results in a condensation threshold of less than 5 μJ cm^–2^, 1 order of magnitude lower than the lasing threshold reported in similar systems in the weak coupling limit.

A Bose–Einstein condensate (BEC) is a system of bosonic (quasi)particles that have undergone thermalization to occupy the ground state. The phase transition to form a BEC occurs at a critical temperature that depends on the effective mass of the bosons. Condensation of exciton–polaritons (EPs) has been widely investigated over the past decades.^1−7^ EPs are bosonic quasi-particles that result from the strong coupling of photons in an optical cavity and excitons in a semiconductor.^8,9^ Due to their low effective mass, EPs can form condensates at high temperatures and even at room temperature when the EP binding energy is sufficiently large. EPs in a condensate occupy the same quantum state. Therefore, when EPs decay, emitting radiation, this radiation may leak from the cavity, producing coherent emission, known as polariton lasing. Polariton lasers do not require population inversion, which potentially enables lasing at much lower thresholds than conventional lasers.^3^ Therefore, EP condensates offer a promising alternative to achieve continuous wave and electrically driven laser-like emission from solid-state organic devices.

The first demonstration of EP condensation was realized with inorganic quantum wells in a Fabry–Perot microcavity at cryogenic temperatures.^1^ Subsequent research has shown that strong light–matter coupling with excitons in organic semiconductors, which have much larger binding energies than excitons in inorganic materials, can also lead to EP condensation at room temperature.^2,4^ While most polariton condensation experiments have been conducted in Fabry–Perot microcavities, this phenomenon has also been observed in optical metasurfaces supporting the so-called surface lattice resonances (SLRs).^5,10^ The latter offers the advantage of easy fabrication over large areas and possible application in integrated photonics. In contrast to a gas of atoms, the original platform for BEC,^11^ exciton polaritons have very short lifetimes. These short lifetimes limit the buildup of the EP density at the ground state, which results in an increased threshold for condensation. Consequently, EP condensation requires powerful laser systems to produce a sufficiently high number of excitons and reach the threshold, which makes polariton lasing unsuitable for most applications.

In this paper, we demonstrate low threshold EP condensation by significantly reducing the losses in an all-dielectric cavity formed by a silicon (Si) metasurface, thus increasing the EP lifetime. Recent efforts have succeeded to reduce the condensation thresholds by replacing metallic metasurfaces supporting plasmonic SLRs with low-loss dielectric metasurfaces supporting Mie-SLRs.^12^ Due to the high *Q*-factors (400–700) of the SLRs, partly due to the reduction of material losses, the condensation threshold was reduced significantly. Here, we explore the limits of organic EP condensation by suppressing radiation losses. This suppression is achieved by coupling excitons to symmetry-protected bound states in the continuum (BICs) supported by an array of Si Mie resonators. BICs are optical modes with infinitely long lifetimes in lossless surfaces due to cancellation of radiation leakage. This suppression of radiation leakage is imposed by the symmetry mismatch between the mode profiles at the surface and those of the radiation continuum, which results in a vanishing of the overlap integral.^13^ Zhen et al. showed that these modes are associated with a topological charge, making them robust to perturbations and visible in the far-field as a polarization vortex.^14^ By the strong coupling of excitons to BICs in a dielectric metasurface, we reduced the EP condensation threshold to 5 μJ cm^–2^.

The lack of radiative losses in BICs, despite the fact that these are modes in the radiation continuum, has recently attracted significant research interest in various fields. BICs are a promising platform for photon lasing, which has been demonstrated for various gain media, such as quantum wells,^15−17^ quantum dots,^18−20^ and organic materials,^21−24^ or with the semiconducting metasurface itself as gain medium.^25^ These systems are in the weak light–matter coupling regime, thus corresponding to conventional photon lasers, where population inversion is required to achieve a net gain and lasing action. However, in the strong light–matter coupling regime, it is possible to reach polariton condensation and coherent emission without population inversion and at potentially lower thresholds. Strong light–matter coupling with BICs has been observed for several systems,^26−30^ but only very recently has polariton condensation been reported in a system of GaAs quantum wells at cryogenic temperatures.^6,7^ The low exciton binding energies in inorganic semiconductors, typically below the thermal energy at room temperature, make low temperatures necessary. To overcome this limitation, we designed dielectric metasurfaces supporting BICs and couple them to organic molecules. We reach the strong light–matter coupling regime and achieve EP condensation from a BIC at room temperature and low thresholds. This result sets an important step forward toward the realization of electrically driven coherent emission from organic systems.

The investigated sample, consisting of a square array of Si nanodisks (height *h* = 90 nm, diameter *d* = 90 nm, lattice constant *P* = 420 nm) on top of a quartz substrate, is represented schematically in Figure 1a. Arrays of polycrystalline Si nanodisks were fabricated using electron beam lithography as described in Methods, the Supporting Information section S8, and illustrated by the scanning electron microscopy (SEM) image shown in Figure 1b. The polycrystalline nature of the particles results in lower material losses compared to amorphous silicon, which is essential for a low condensation threshold.

**Figure 1:**
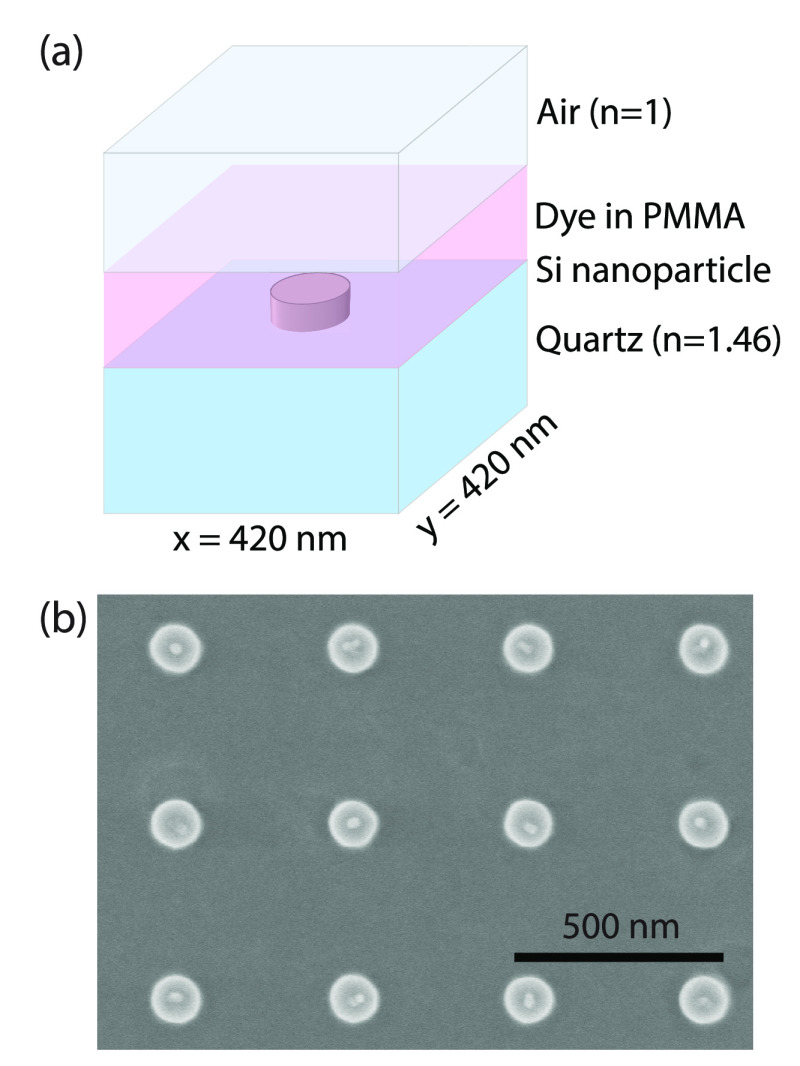
(a) Schematic overview of a unit cell of the Si metasurface covered with a 200 nm layer of perylene dye in PMMA. (b) Top view SEM image of the Si nanoparticle array.

When the array is embedded in a medium with a homogeneous refractive index, the Mie resonances of the individual nanoparticles can couple radiatively through in-plane diffractive orders, resulting in a forward and backward propagating transverse electric (TE) Mie SLR (TE-SLR) and a degenerate transverse magnetic TM-SLR (see Supporting Information, section S1).^31^ If the Si metasurface is covered with a higher refractive index thin film (polystyrene film with a thickness of 230 nm and refractive index *n* = 1.59), the Mie resonances can couple the incident light into quasi-guided modes in the film.^32,33^ This coupling is more apparent at high energies in the angle dependent extinction measurements and simulations using the Rigorous Coupled-Wave Analysis (RCWA) method shown in Figure 2a (see Supporting Information S8 for details about measurements and simulations). The two TE-SLRs with an anticrossing at normal incidence^34^ are the dominant modes in this angle dependent dispersion, but additionally two weaker “parabolic” modes are visible, which correspond to the TM-SLR and TM quasi-guided mode. Interestingly, two of these modes become extremely narrow approaching normal incidence and vanish completely at normal incidence, as can be clearly appreciated in the extinction measurements at the angles of incidence of θ = 0° and 0.2° shown in Figure 2b. This characteristic indicates that the SLRs at large wavenumbers evolve into symmetry-protected BICs at θ = 0°.

**Figure 2:**
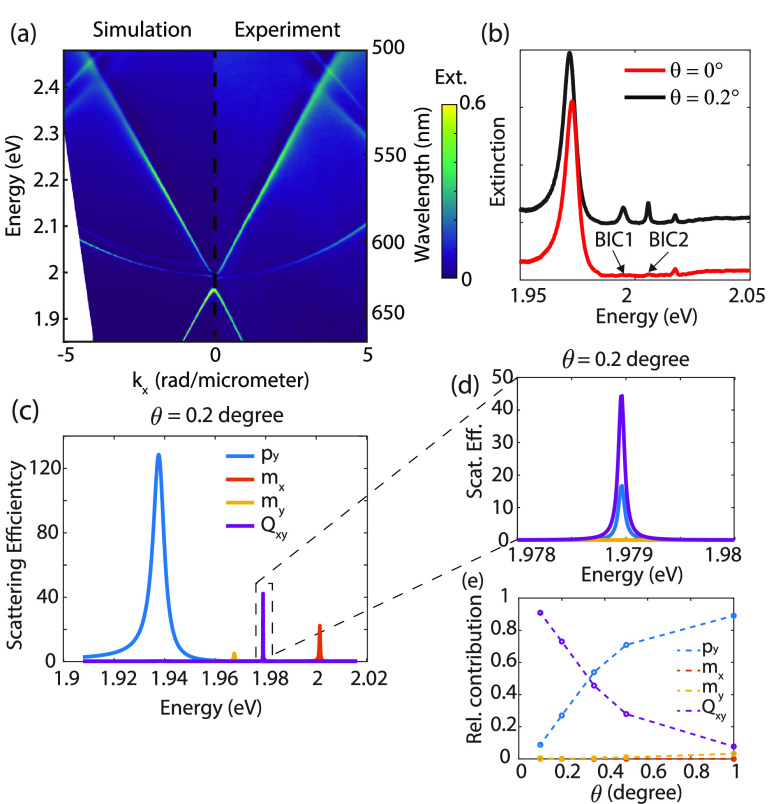
(a) Simulated (left panel) and experimental (right panel) extinction (1-transmission) spectra of the silicon metasurface with a 230 nm thick layer of polystyrene (*n* = 1.59) on top as a function of the parallel component of the wave vector of the incident wave, i.e., , with θ the angle of incidence. (b) Measured extinction spectra at θ = 0° and θ = 0.2°. The energies of the two BICs at normal incidence are indicated. (c) Multipolar decomposition of the resonances for a plane wave incident at 0.2°. (d) Magnified view of (c). (e) Angle dependent contributions of the different multipoles for BIC 2 (i.e., the mode that becomes a BIC at *k* = 0 at 1.979 eV).

To understand the character of these BICs, we have performed a multipolar decomposition of the scattering efficiency by the resonances at different energies for the array covered with a dye doped layer. Using this method, we retrieve the character of the resonances in terms of the electric dipolar, magnetic dipolar, and electric quadrupolar modes (see Methods for details). The result of the decomposition for an incident plane wave at an angle of 0.2° is shown in Figure 2c. The mode at ∼1.94 eV (blue peak in Figure 2c) corresponds to an electric dipolar mode along the *y*-direction, while the mode at ∼2 eV (red peak) corresponds to a magnetic dipolar mode along the *x*-direction. The two modes that become a BIC at normal incidence at ∼1.965 eV (yellow peak, BIC 1) and 1.979 eV (purple peak, BIC 2) have dominant magnetic dipolar and electric quadrupolar character, respectively. The decomposition of BIC 2 is shown in more detail in Figure 2d (for details about the decomposition of the other modes, see section S2 in the Supporting Information). Besides the quadrupolar contribution, there is a dipolar contribution as well, leading to the excitation of the mode at nonzero angles. Figure 2e shows how the character of BIC 2 changes from dipolar at large angles to quadrupolar toward normal incidence. This explains why at normal incidence all radiation losses are suppressed as a pure quadrupolar mode in an infinite lattice can neither be excited from the far field, nor radiate into the far field. The full suppression of radiation losses of the BICs will be exploited for low-threshold polariton condensation when the resonances in the metasurface are coupled to excitons in a film of organic molecules.

To investigate the formation and condensation of EPs, we remove the transparent polystyrene film on top of the array and spin coat a solution of 32 wt % perylene dye ([*N*,*N*′-bis(2,6-diisopropylphenyl)-1,7- and -1,6-bis (2,6-diisopropylphenoxy)perylene-3,4:9,10-tetracarboximide]) in poly(methyl methacrylate) (PMMA) to form a 200 nm thick layer. The bare molecules show two exciton peaks at 2.24 and 2.41 eV, corresponding to the electronic transition and its first vibronic replica, respectively. These peaks are visible in the extinction spectrum plotted with the black curve in the right panel of Figure 3a. The green dashed curve in the same panel shows the normalized photoluminescence (PL) emission spectrum from the layer when excited by a 400 nm laser with a repetition rate of 1 kHz at low fluence, and the red curve corresponds to the normalized amplified spontaneous emission (ASE) spectrum measured at a fluence of >500 μJ cm^–2^.

**Figure 3:**
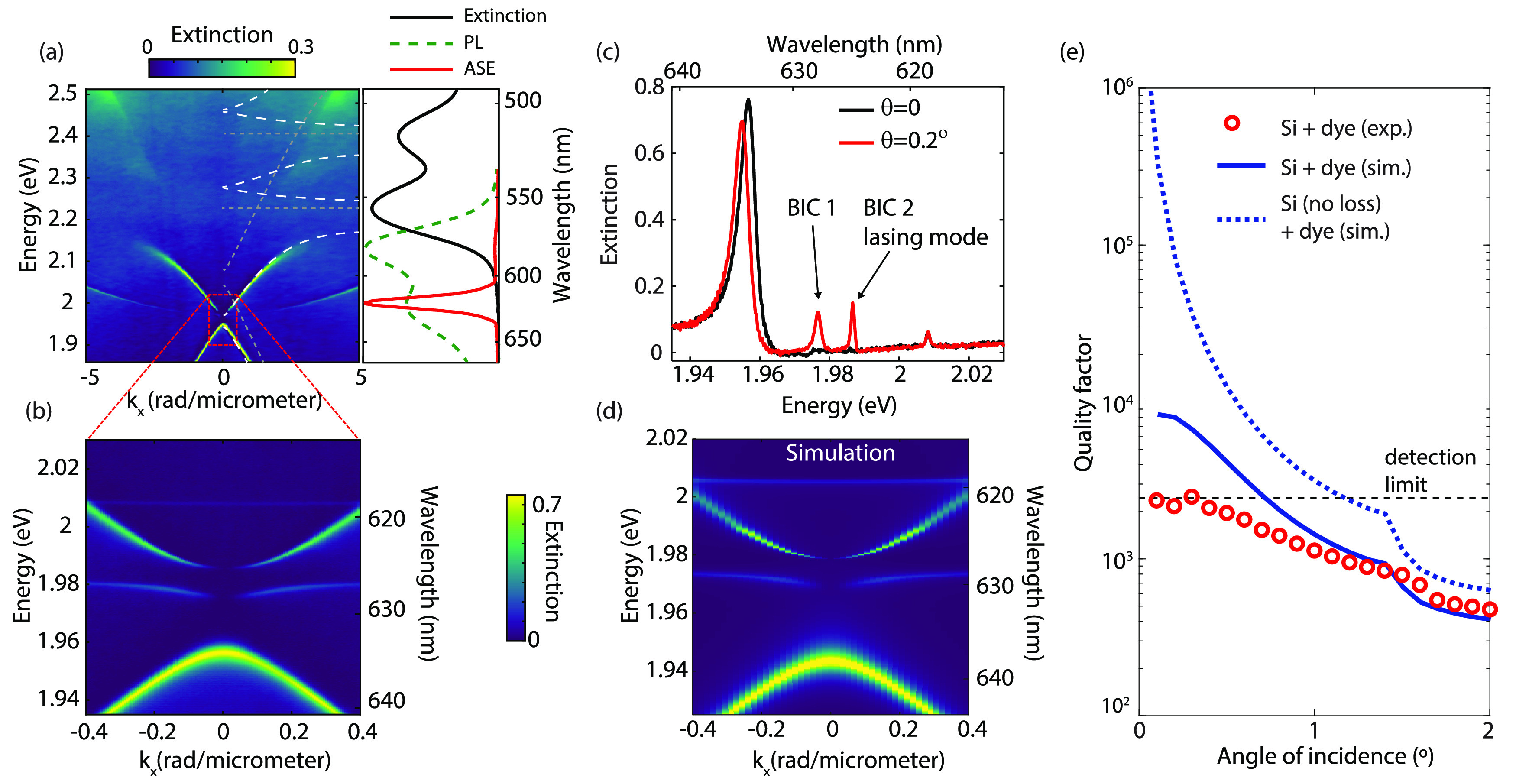
(a) Optical extinction of the array of Si nanodisks as a function of the in-plane momentum (*k*_*x*_) measured with a Fourier microscope. The gray dashed lines indicate the energies of the bare SLRs and the exciton energies. The white dashed curves are the EP dispersion resulting from the coupling of the SLRs with the excitons, as calculated with a 4-state coupled oscillator model. The right panel of (a) shows the extinction, photoluminescence, and amplified spontaneous emission of the bare layer of perylene dye in PMMA. (b) Magnified view of (a), where two BIC modes are dark at normal incidence. (c) Extinction spectra measured at θ = 0° and 0.2°, showing two narrow modes for θ = 0.2°. (e) Quality factor of the modes turning into BIC 2, as a function of the angle of incidence. The red circles indicate the *Q*-factor calculated from the measured width of the resonance, the blue curve shows the simulated *Q*-factor for the resonance for silicon nanoparticles with realistic losses, and the blue-dashed curve shows the *Q*-factor for Si without losses.

A high concentration of molecules in the film is required to achieve collective strong light–matter coupling, as the coupling strength scales with the square root of the number of dye molecules in the mode volume, i.e., *g* = *g*_0_√*N*, where *g*_0_ is the coupling strength for a single molecule in the cavity, *N* is the number of molecules and *g* is the collective coupling strength.^35^ We note that for a 32 wt % perylene dye concentration in PMMA, the emission quantum efficiency is below 5%,^5^ which is detrimental for photon lasing in the weak-coupling regime, but is not a limitation for polariton lasing in the strong-coupling regime. The collective strong coupling of the molecules and the lattice resonances is manifested in the momentum-resolved transmission measurement as an anticrossing of the exciton resonance and the TE-SLRs, as shown in Figure 3a. The dispersion of the resulting EPs can be fitted by a four-level coupled oscillator model incorporating the forward and backward propagating TE-SLRs and two exciton resonances^36^ (details of the coupled oscillator model including the Hopfield coefficients of the LPB are given in section S3 of the Supporting Information). The energies of these exciton transitions and the dispersion of the bare cavity modes are plotted with the gray dotted lines in Figure 3a (for *k*_*x*_ > 0), and the resulting coupled resonances are shown with the white-dashed curves. Note that in the coupled oscillator model we do not include the TM-SLR and quasi-guided modes as these modes are much less relevant in the dispersion compared to the TE-SLRs. At normal incidence (*k*_*x*_ = 0), the EPs energies overlap with the ASE, indicating that these polaritons are in the region with the highest gain in the film.^37^ We note also that the energy difference between the exciton–polariton reservoir (2.24 eV) and the BIC (1.99 eV) is approximately equal to the energy difference between the exciton electronic transition and the vibronic replica (2.41 eV), i.e., ≃200 meV. As shown previously,^5^ these similar energy differences allow an efficient vibronic-assisted relaxation of polaritons from the reservoir to the bottom of the lower polariton band to form the condensate.

We zoom in further in the dispersion measurements to the region around *k*_*x*_ = 0 by using a low numerical aperture (NA) objective (Nikon 10x, 0.3 NA) and a finer grating in the spectrometer (600 lines/mm). We observe that, similar to the array covered with polystyrene, two of the four modes show an increasing quality factor toward normal incidence, eventually becoming dark at *k*_*x*_ = 0 (see Figure 3b). The measured dispersion matches excellently with the RCWA simulations (Figure 3d). The narrow line width at small angles of incidence becomes especially clear in the spectra taken at θ = 0.2° and normal incidence, as plotted with the red and black curves in Figure 3c. From the spectrum at θ = 0.2°, there is a difference in the line width between the mode at 1.977 eV (BIC 1, magnetic dipolar character) and the mode at 1.987 eV (BIC 2, quadrupolar character), where the latter clearly has a higher *Q*-factor (see the Supporting Information, section S4, for a detailed analysis of the fields and *Q*-factors of both modes).

To investigate the properties of BIC 2 in more detail, we fit the resonance width with a Fano line shape^38,39^
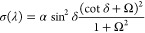
1where , λ_0_ is the resonance wavelength, Γ is the line width, δ is the Fano parameter and α is the amplitude of the resonance. From the line width and resonance wavelength, we calculate the *Q*-factor as *Q* = λ_0_/Γ. The resulting *Q*-factor as a function of the angle of incidence obtained from the experimental transmission spectra is plotted with red circles in Figure 3e. The *Q*-factor increases toward smaller angles until it reaches a *Q*-factor of around 2200. As expected for any real system with unavoidable imperfections and a limited size, the *Q*-factor does not diverge. However, an experimental *Q*-factor of 2200 is among the highest reported for Mie metasurfaces in the visible range.^40^

We compare the experimental *Q*-factor with the *Q*-factor extracted from the RCWA simulations by fitting the simulated dispersions with the Fano profile (eq 1). The blue curve in Figure 3e shows the calculated *Q*-factors for the simulated array of Si nanorods with a permittivity accounting for the losses in the silicon nanoparticles. These losses have been estimated in previous measurements to increase the imaginary component of the permittivity of polycrystalline Si by a factor of 5 compared to crystalline Si.^12^ The simulated *Q*-factor follows a trend similar to the experimental one but reaches a value of approximately 10^4^ at small angles. At this point, material losses limit further increase of the *Q*-factor. If we set these losses equal to zero in the RCWA simulations, the *Q*-factor indeed diverges when approaching *k*_*x*_ = 0, as expected for a symmetry-protected BIC (dotted curve in Figure 3e).

If we pump the system nonresonantly with an amplified laser system (Vitarra, λ = 400 nm, repetition rate = 1 kHz, pulse duration ∼150 fs) at low fluences, we can resolve the *k*_*x*_ and energy dependent fluorescent emission of the sample (see Figure 4a). This emission is clearly dominated by the decay into the different modes and the outcoupling into the far-field by the metasurface, with the strongest emission from the lower polariton band (BIC 2). This process alters completely the emission spectrum of the bare molecules. When the pump fluence is increased to approximately *P*_*th*_ = 5 μJ cm^–2^, the emission mostly originates from two points in the *k*-space around 1.987 eV, indicating that the polariton lasing threshold is reached (Figure 4b). At higher pump fluences of 1.2 *P*_*th*_ the emission is fully dominated by this coherent emission (Figure 4c). Since the BIC itself is nonradiative at *k*_*x*_ = 0, a vortex beam in reciprocal space is formed with the light being emitted from angles slightly off normal incidence. Further polarized analysis of the emission as a function of *k*_*x*_ and *k*_*y*_, shows the vortex behavior of the emission(Figure 4d), experimentally evidencing the topological character of the BIC^14^ and its quadrupolar nature.^20,24^

**Figure 4:**
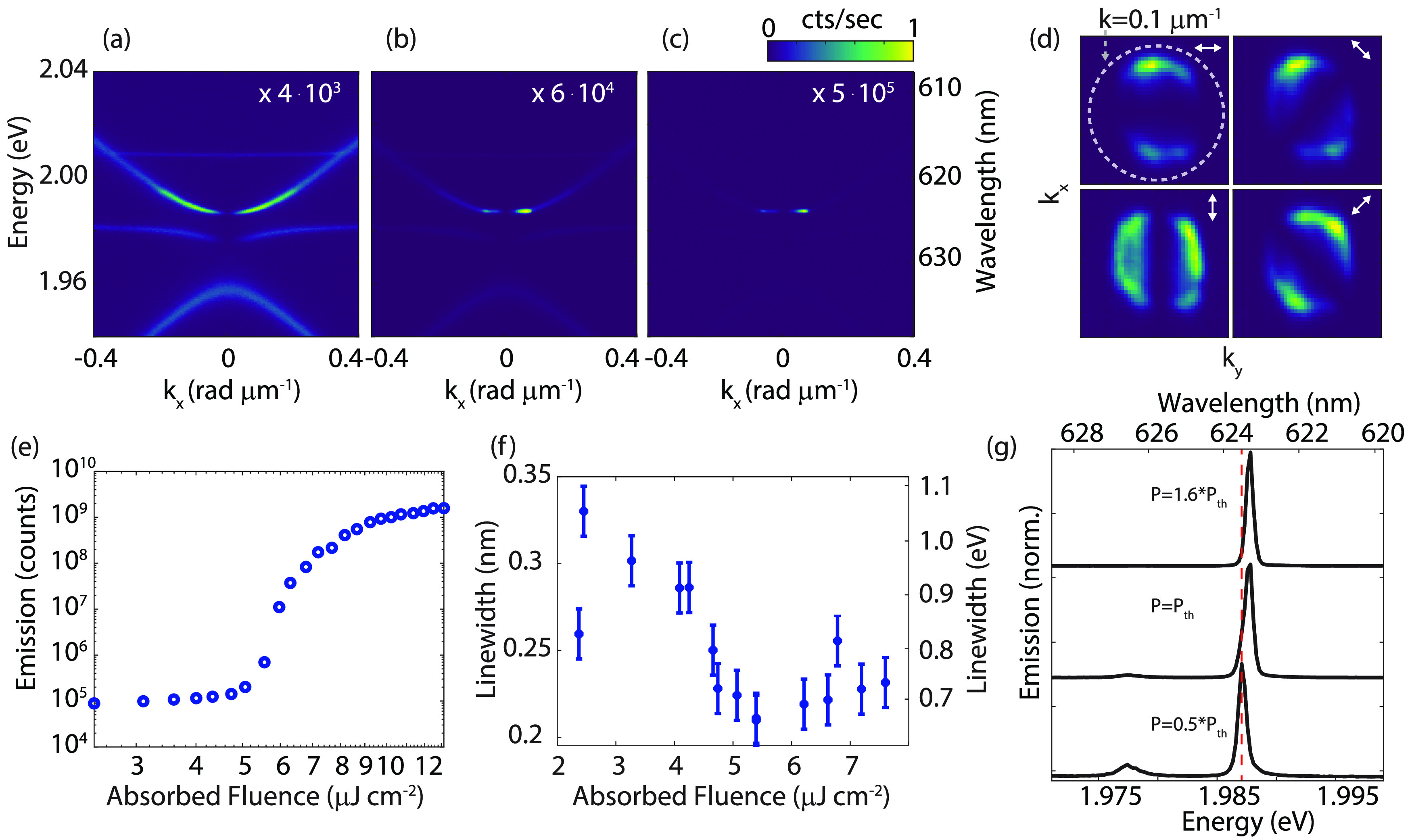
Emission spectra as a function of in-plane momentum for different pump fluences: (a) below threshold, (b) on threshold, and (c) above threshold. (d) Coherent emission for an excitation power of 1.2*P*_*th*_ as a function of *k*_*x*_ and *k*_*y*_, evidencing the vortex behavior. (e) Emission intensity versus pump fluence integrated over *k*_*y*_ and *k*_*x*_ from −0.15 to 0.15 rad μm^–1^. (f) Full-width at half-maximum (FWHM) of the emission as a function of pump fluence. (g) Emission spectra below and above the threshold, showing the blue shift of the emission peak as the pump fluence is increased.

We monitor the nonlinear emission by plotting in Figure 4e the integrated emission over a solid angle from *k*_*x*_,*k*_*y*_ = −0.15 to *k*_*x*_,*k*_*y*_ = 0.15 μm^–1^, evidencing the condensation threshold at *P*_*th*_ = 5 μJ cm^–2^. A second threshold at higher fluences indicated that stimulated emission could not be observed in the range of pump fluences that can be used without damaging the sample. This is an expected result due to the low quantum efficiency of the molecular layer at high concentrations.^5^ We further confirm the transition from spontaneous emission to polariton lasing and the formation of the EP condensate by measuring the reduction in the line width, as plotted in Figure 4f for *k*_*x*_ = 0.05 μm^–1^. This line width reduction is a clear indication of the increased temporal coherence of the condensate. Finally, Figure 4g illustrates the blue shift of the emission peak as the pump fluence increases. Similar blue shifts have been observed in organic condensates and explained by polariton–polariton interactions,^4,41^ although alternative explanations to this blue shift have been also proposed.^42^

We note that the observed condensation threshold of 5 μJ cm^–2^ is 40% lower than previously reported values from SLRs in similar arrays of Si nanoparticles,^12^ the lowest reported condensation threshold being at room temperature. A major difference with ref (12) is that condensation in that work takes place in the bright SLR. The photonic character of the lower polariton band at the BIC energy, due to the detuning with respect to the electronic transition energy of the excitons, and the inherited low radiation losses of the polaritons at this energy favor the reduction of the condensation threshold. This threshold is comparable to the recent results at cryogenic temperatures in inorganic semiconductors, illustrating the potential of BICs in metasurfaces for room-temperature condensation in organic systems at ultralow thresholds.

In conclusion, we have demonstrated room-temperature exciton–polariton condensation from a BIC in a metasurface of Si nanoparticles resulting from SLRs. The lack of radiative losses and the low material losses of Si result in very high quality factors of the polariton mode. The condensation threshold of 5 μJ cm^–2^ is the lowest reported threshold for an organic small molecule polariton condensate and comparable to values measured at low temperatures in inorganic systems. These results set an important step toward the realization of room-temperature electrically pumped organic polariton lasers, which require low thresholds.

## Methods

The multipole decomposition of the observed modes was performed with the Electromagnetic Waves in the Frequency Domain module of COMSOL Multiphysics. Details on the simulations can be found in Supporting Information S4.

For the simulated dispersion of the dielectric metasurfaces we used rigorous coupled-wave analysis (RCWA) based on refs (43−45). For a more detailed description, see Supporting Information S7.

## Supporting Information Available

Simulation and measurements of the metasurface in a homogeneous environment, the mode decomposition of the four resonances, description of the coupled oscillator model, *Q*-factors of the two BIC modes, COSMOL simulations, the Fourier microscope, RCWA method, details on the fabrication of the silicon metasurface, dispersion for P-polarized light, and the visualization of the condensation in momentum (*k*_*x*_, *k*_*y*_) space (PDF)
