# Supplementary material for: Room Temperature Exciton–Polariton Condensation in Silicon Metasurfaces Emerging from Bound States in the Continuum
Source: Nano Lett. 2023 Jun 13;23(12):5603–9. doi: 10.1021/acs.nanolett.3c01102 (PMC10311526; doi:10.1021/acs.nanolett.3c01102)
Supplement: Supplementary file 1 — nl3c01102_si_001.pdf [file nl3c01102_si_001.pdf]

Supporting Information for "Room Temperature Exciton-Polariton Condensation in Silicon Metasurfaces Emerging from Bound States in the Continuum"

Anton Matthijs Berghuis[1,2], Gabriel W. Castellanos[1,2], Shunsuke Murai[3], Jose Luis Pura[4,5], Diego R. Abujetas[6], Erik van Heijst[1,2], Mohammad Ramezani[1,2], José A. Sánchez-Gil[4], and Jaime Gómez Rivas[1,2]

[1]Department of Applied Physics and Science Education and Eindhoven Hendrik Casimir Institute, Eindhoven University of Technology,, P.O. Box 513, 5600 MB Eindhoven, The Netherlands.
[2]Institute for Complex Molecular Systems-ICMS, Eindhoven University of Technology, P.O. Box 513, 5612 AJ, Eindhoven, The Netherlands
[3]Department of Material Chemistry, Graduate School of Engineering, Kyoto University, Katsura, Nishikyo, 6158510, Kyoto, Japan.
[4]Instituto de Estructura de la Materia (IEM-CSIC), Consejo Superior de Investigaciones Científicas, Serrano 121, 28006 Madrid, Spain.
[5]GdS-Optronlab, Física de la Materia Condensada, Universidad de Valladolid , Paseo de Belén 19, 47011 Valladolid, Spain
[6]Physics Department, Fribourg University, Chemin de Musée 3, Fribourg 1700, Switzerland.

Contents

S1 Modes Supported by a Periodic Array in a Homogeneous Environment S2

S2 Multipolar Decomposition of All Modes S3

S3 Coupled Oscillator Model S4

S4 Q-factors of the BICs S6

S5 COMSOL Simulations S7

S6 Fourier Microscope Design S7

S7 Rigorous Coupled-Wave Analysis Simulations S8

S8 Fabrication of the Silicon metasurface S8

S9 Dispersion for P-polarized Light. S9

S10 Condensation in Momentum Space S10

S1 Modes Supported by a Periodic Array in a Homogeneous Environment

When the Si metasurface is embedded in a homogeneous medium (n=1.46), the sample has a distinct response from the sample covered with a higher refractive index medium (right panel of Fig. S1a). Two linear disperions are visible, associated with the TE SLRs. The parabolic shaped dispersion visible in the same figure is associated with a magnetic SLR. The simulated spectrum is similar to an angle dependent extinction measurement of s-polarized light of the sample covered with a 200 nm PMMA layer, resulting in an approximately homogeneous environment (Fig. S1a). The extinction at an angle of 0.2°, displayed by the black curve in Fig. S1b, indeed shows three peaks of which one vanishes at normal incidence (red curve in Fig. S1b).

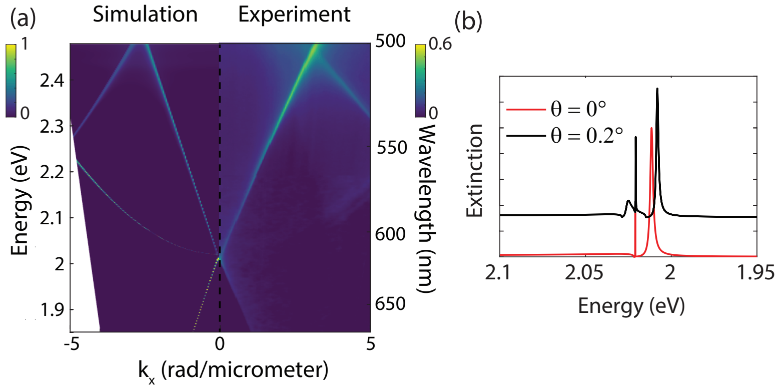

Figure S1: (a) Left panel: dispersion of the array covered with a 200 nm PMMA layer, right panel: RCWA simulation of the array in a homogeneous environment. (n=1.46). (b) cross sections of the simulation in (a) at 0° (red curve) and 0.2° (black curve)

S2 Multipolar Decomposition of All Modes

Multipolar decompositions of the 4 modes in the Si metasurfaces at an incident angle of 0.2° are shown in Fig. S2 (a-d). The spectra are decomposed in terms of the electric dipoles along the y-direction (p_y), magnetic dipole along the x-direction (m_x) and y-direction (m_y), and the quadrupolar modes (Q_{xy} and Q_{yz}) The angle dependent contributions of the different multipoles to the corresponding modes are shown in Fig. S2 (e-f).

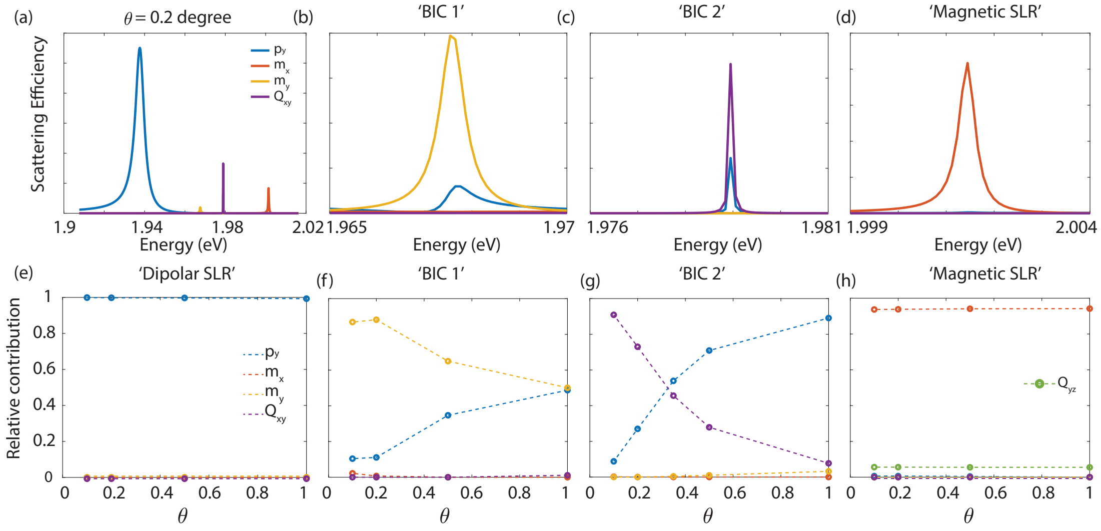

Figure S2: (a-d) Multipolar decompositions of the 4 modes. (e-g) Angle dependent contributions of the different multipoles to the corresponding modes

S3 Coupled Oscillator Model

The polariton dispersion is fitted by applying a coupled oscillator model twice. First, the SLR dispersions are computed from the coupled oscillator model of the Rayleigh Anomalies (RAs, in-plane diffractive orders) with the Mie-resonances of the individual particles. Subsequently, the polariton dispersion is fitted by applying the coupled oscillator model to the SLR dispersions and the exciton resonances. The measured SLRs dispersion can be described by the eigenvalues of the following Hamiltonian:

$$H = \begin{bmatrix} E_{Mie} - i\frac{\gamma_{Mie}}{2} & g_1 & g_2 \\ g_1 & E_{RA1} - i\frac{\gamma_{RA1}}{2} & 0 \\ g_2 & 0 & E_{RA2} - i\frac{\gamma_{RA2}}{2} \end{bmatrix} \tag{1}$$

where $\gamma_{Mie} = 100$ meV is the loss of the Mie-resonance extracted from the FWHM of the localized resonance; $g_1 = g_2 = 100$ meV are the coupling constants between the forward and backward propagating TE-RAs and the Mie resonances. $\gamma_{RA} = 2$ meV are the losses of the RAs, and the energy of the Mie resonances (E_{Mie}) is 2.6 eV. The energy of the TE-RA depends on the incident wave vector and is calculated as:

$$E_{RA}(k_x) = \mp\frac{c}{n_{eff}}[k_x \mp \frac{2\pi}{a_x}], \tag{2}$$

with c the speed of light in vacuum, k_x the in-plane momentum along the x-direction, and $a_x = 420$ nm the period of the lattice in the x-direction. $n_{eff} = 1.42$ is the effective refractive index of the surrounding medium, which is slightly lower than the index of the quartz substrate ($n = 1.46$) because of the small thickness of the layer on top of the array. These energies (E_{SLR1}, E_{SLR2}), obtained for the SLRs are then used to fit the dispersion of the polaritons with a 4-level coupled oscillator model, incorporating the two TE-SLRs and the two excitonic transitions in the dye at E_{excl}=2.41 and E_{exc2}=2.24 eV.

$$H = \begin{bmatrix} E_{SLR1} - i\frac{\gamma_{SLR1}}{2} & 0 & g_C & g_C \\ 0 & E_{SLR2} - i\frac{\gamma_{SLR2}}{2} & g_C & g_C \\ g_C & g_C & E_{exc1} - i\frac{\gamma_{exc1}}{2} & 0 \\ g_C & g_C & 0 & E_{exc2} - i\frac{\gamma_{exc2}}{2} \end{bmatrix} \tag{3}$$

Here, $g_C = 270$ meV, is the coupling strength between the exciton transitions and the SLRs. γ_{SLR1} and γ_{SLR2} are the losses of the SLRs as obtained from Eq. (1), and γ_{exc1}=γ_{exc2}=140 meV the losses of the two exciton transitions. At the point where exciton 1 and the SLR cross, this corresponds to a value of 25 meV. The results of the model are plotted in Fig. 3 of the main text and plotted again for the LPB in Fig. S3 a. The Hopfield coefficients of the LPB are shown Fig. S3 b. At the Γ point, the total exciton fraction of the LPB is approximately 20%.

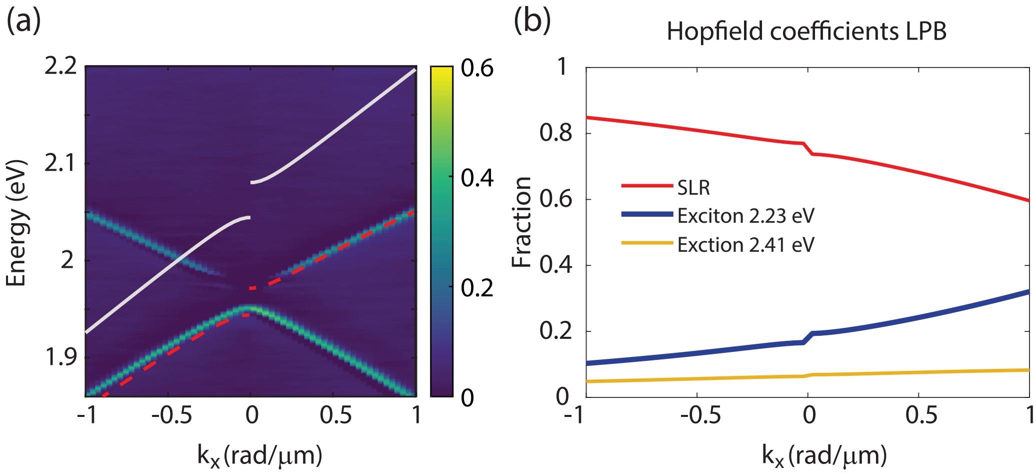

Figure S3: (a) Experimental dispersion of the cavity mode. The white curve is a simulation of the uncoupled SLR mode, the red dashed curve indicates the LPB energy obtained from the coupled oscillator model. (b) Shows the SLR fraction and exciton fractions of the lower polariton. At the Γ point, the total exciton fraction is approximately 20 %

S4 Q-factors of the BICs

Due to the different field distributions of the two BICs, the Q-factors evolve differently as a function of the in-plane wave vector. Figure S4a. shows the dispersion these modes. the vertical cross sections of a. at the energies of BIC 1 and BIC 2 are plotted with the black dots in Fig. S4b. and (c) respectively. The peaks are fitted with a Fano profile, with are plotted with the colored corves in (b) and (c). From the width of the Fano profiles, the Q-factors are obtained and plotted in Fig. S4. The blue circles give the experimental Q-factors and the blue curve the simulated Q-factor for BIC 2, where the losses in the silicon are implemented. The Q-factor increases rapidly when approaching normal incidence. BIC 1 on the other hand has a relatively high Q-factor at large angles, but the value is nearly constant over the range of wave vectors, as shown with the red circles and red curve in Fig. S4d.

The difference between the Q-factors of BIC 1 and BIC 2 can be explained from the electric near-field distributions in the XY-plane of the sample for different angles of incidence, plotted in Fig. S4e for z=45 nm, i.e. a height of 45 nm above the glass-particle interface. The fields of BIC 1 at 0.1° have a higher fraction inside the particles compared to BIC 2, explaining the larger losses of BIC 1 at small angles. With increasing angles, BIC 2 evolves into a more dipolar mode with TE character while BIC 1 retains a of magnetic character, resulting in lower radiative losses.

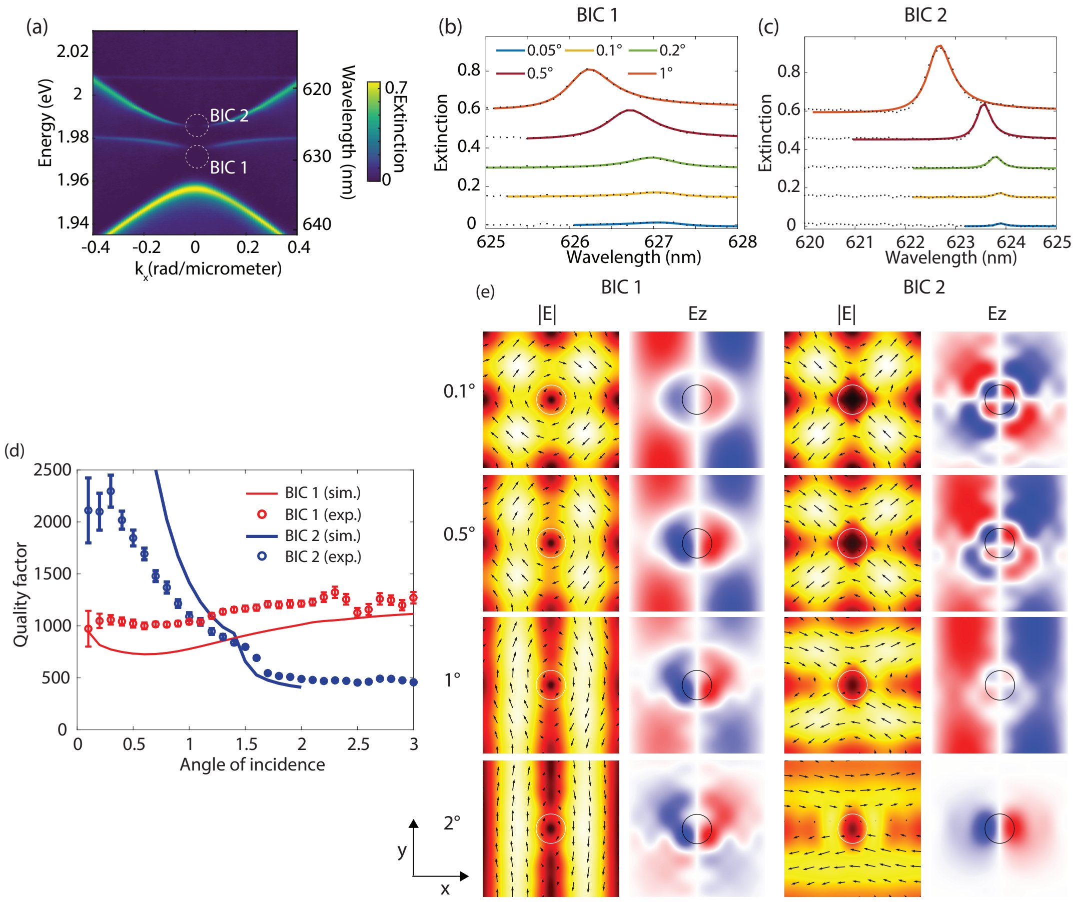

Figure S4: (a) Measured dispersion of the sample. The cross sections of (a) at the wavelengths around BIC 1 and BIC 2 are plotted in (b) and (c) respectively. The data is plotted with the black dots and the Fano fits by colored solid curves. From the width of the Fano fits the Q-factor is determined and plotted in (d). (e.) simulated electric fields of BIC 1 and BIC 2 at small angles.

S5 COMSOL Simulations

The multipole decomposition of the observed modes was performed with the Electromagnetic Waves in Frequency Domain module of COMSOL Multiphysics. The simulation was done for the array covered with the rylene dye molecules in PMMA, using the same material properties and configuration of the RCWA simulations, we calculated the polarization **P** induced in the system by external plane waves impinging at different angles with TE polarization. The multipole decomposition of the modes can be calculated by integrating **P**:[1,2]

$$\mathbf{p} = \int \mathbf{P} d\mathbf{r} \; , \tag{4}$$

$$\mathbf{m} = -\frac{i\omega}{2} \int (\mathbf{r} \times \mathbf{P}) \mathbf{r} d\mathbf{r} \; , \tag{5}$$

$$\overline{\overline{\mathbf{Q}}} = 3 \int (\mathbf{r}\mathbf{P} + \mathbf{P}\mathbf{r}) d\mathbf{r} \; . \tag{6}$$

The juxtaposed terms, such as **rP**, represent the dyadic product of both vectors. The integrals are calculated over the volume of the scatterers (i.e., the Si nanodisks) using its geometric center as the coordinate origin. Note that on this basis, the electric quadrupole tensor is totally symmetric and not traceless, and the toroid-dipole terms would not appear. The contribution of the magnetic quadrupole and superior orders have been confirmed to be negligible. Once the multipolar terms are known, we can compute the scattering efficiency of each one with

$$Q_{sca}^{ED_i} = \frac{k_0^4}{6\pi^2 \varepsilon_0^2 E_0^2 R^2} |p_i|^2 \; , \tag{7}$$

$$Q_{sca}^{MD_i} = \frac{\eta_0^2 \varepsilon_d k_0^4}{6\pi^2 E_0^2 R^2} |m_i|^2 \; , \tag{8}$$

$$Q_{sca}^{EQ_{ij}} = \frac{\varepsilon_d k_0^6}{80\pi^2 \varepsilon_0^2 E_0^2 R^2} |Q_{ij}|^2 \; , \tag{9}$$

where $i, j = x, y, z$, η_0 is the vacuum impedance, and R is the nano-disks' radius.

S6 Fourier Microscope Design

To map the energy and momentum resolved extinction of the metasufaces, we use a Fourier microscope in transmission mode. The excitation objective (40x, 0.6NA) focuses a white light onto the sample. The transmitted light is collected by a 60x, 0.7 NA or 10x, 0.3 NA objective. The back focal plane of this objective is imaged on the spectrometer slit (Princeton Instruments SP2300) using 2 lenses in a 4f configuration (see Fig. S5). The spectrometer transmits light along one of the principal axis of the metasurface (k_x or k_y) and disperses light on the CCD camera (Princeton Instruments ProEM:512) using a 150 or a 600 lines/mm grating. For fluorescence/lasing measurements, the objective is replaced by an f=10cm lens and a 400 nm laser at 1 kHz is used for excitation. This beam is generated by frequency doubling the output of a Ti:sapphire regenerative amplifier (Coherent Astrella), λ= 800 nm with a pulse duration of 150 fs.

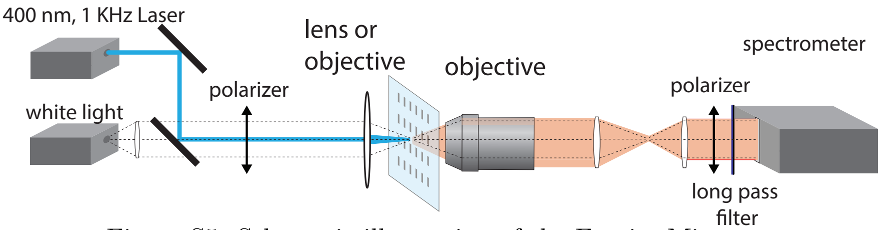

Figure S5: Schematic illustration of the Fourier Microscope

S7 Rigorous Coupled-Wave Analysis Simulations

For the simulated dispersion of the dielectric metasurfaces we used rigorous coupled-wave analysis (RCWA). RCWA is a semi-analytical method that solves Maxwell's equations in Fourier space and offers great simulation speed for dielectric structures. We developed a homemade code based on Refs.[3,4]. In addition to conventional RCWA the Normal Vector Method[5] was applied to increase convergence of the simulations. This allowed the use of 11 x 11 spatial harmonics to accurately describe the unit cell for the calculation of the transmittance of a plane wave through the sample. The permittivity values of Si were taken from literature[6] and the permittivity of the dye-doped layer were obtained from ellipsometry measurements.

S8 Fabrication of the Silicon metasurface

Polycrystalline Si thin films with a thickness of 90 nm were grown on a synthetic silica glass substrate by low-pressure chemical vapor deposition using SiH_4 gas as a source of Si. A resist (NEB22A2, Sumitomo) was cast on the Si film and exposed to electron-beam lithography, followed by development to make nanoparticle arrays of resist on the Si film. The Si film was vertically etched using a selective dry etching (Bosch process) with SF_6 and C_4F_8 gases, and the resist residue was etched away by oxygen dry etching. The fabricated array covered and area of 2.5×2.5 mm^2

S9 Dispersion for P-polarized Light.

The dispersion of the sample for s-polarized light is described in the main text. In this section, the modes for p-polarized light are discussed. The extinction of the sample illuminated by p-polarized light is plotted in Fig. S6a for a 0.6 NA collection objective, and in Fig. S6b for a 0.3 NA collection objective. While there should be two bright modes at normal incidence (because s- and p-polarization are equivalent at normal incidence for a square array), only one mode is clearly visible, which is most likely due to the resolution limit of the spectrometer. When we slightly tilt the sample such that $k_y \simeq 0.2\,\text{rad}\,\mu\text{m}^{-1}$, the two modes that are BICs at $k_y = 0$ become visible in the dispersion (Fig. S6c).

In Fig. S6d, the emission of p-polarized light along k_x is investigated. Due to the width of the spectrometer slit, the emission is integrated over a range of wave vectors in the y-direction. Therefore, we see a signal from the two modes that become a BIC at k_y=0 and the bright mode, instead of only the bright mode. Zooming in further to smaller wave vectors by using the low NA objective and $k_y \simeq 0.2\,\text{rad}\,\mu\text{m}^{-1}$, we resolve the other bright mode at 2.01 eV and two additional BICs at slightly higher ($\sim$ 2.015 eV) and lower energies ($\sim$ 2.005 eV) (Fig. S6e). When we increase the pump fluence, the polariton lasing emission is indeed leaking from the same BIC as for s-polarized light, as visible in the two other panels of Fig. S6e.

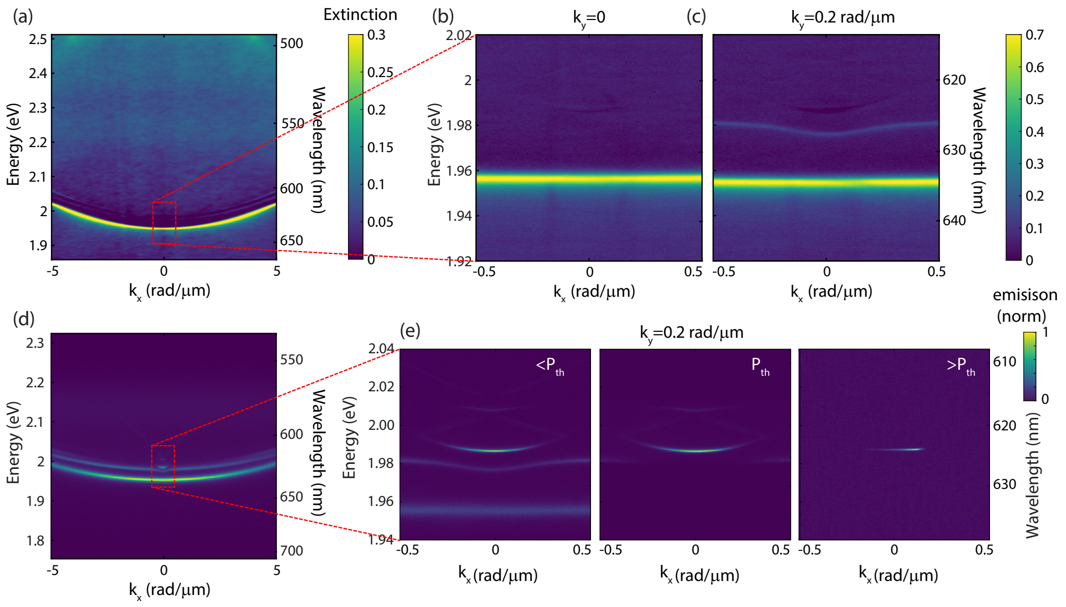

Figure S6: (a) Extinction of the sample with a 200 nm dye layer for p-polarized light. (b) magnified view of (a). When the sample is also slightly tilted along y ($k_y = 0.2$) an additional mode is visible (c). (d) P-polarized emission of the sample. (e) Emission of the p-polarized light below, on and above threshold.

S10 Condensation in Momentum Space

When the spectrometer slit is opened, we can image the 0-order reflection of the grating, resulting in a map of the emission as a function of both k_x and k_y. We put a band pass filter centered at 630 nm with a 10 nm bandwidth (Thorlabs, FBH630-10) in the optical path. In Figure S7a-d, we plot the emission for x-polarized light. In Fig. S7a, the horizontal-curved bands correspond to the broad bright mode in the dispersion. The sharp-bright triangles pointing at k_x=k_y=0 correspond to the mode evolving into BIC 2 and the vertical line corresponds to the mode associated to BIC 1. We zoom in at the area around k=0 (Fig. S7b), where the lack of emission from BIC 2 at normal incidence is clear. Upon increasing the pump power, the emission becomes more dominated by the mode associated to BIC 2 (Fig. S7c) until eventually all of the emission originates from a small area around k_x=k_y=0 (Fig. S7c). In Figs. S7e-h, the same measurements are shown but for the orthogonal polarization, giving equivalent results (but 90° rotated) due to the C_4 symmetry of the system.

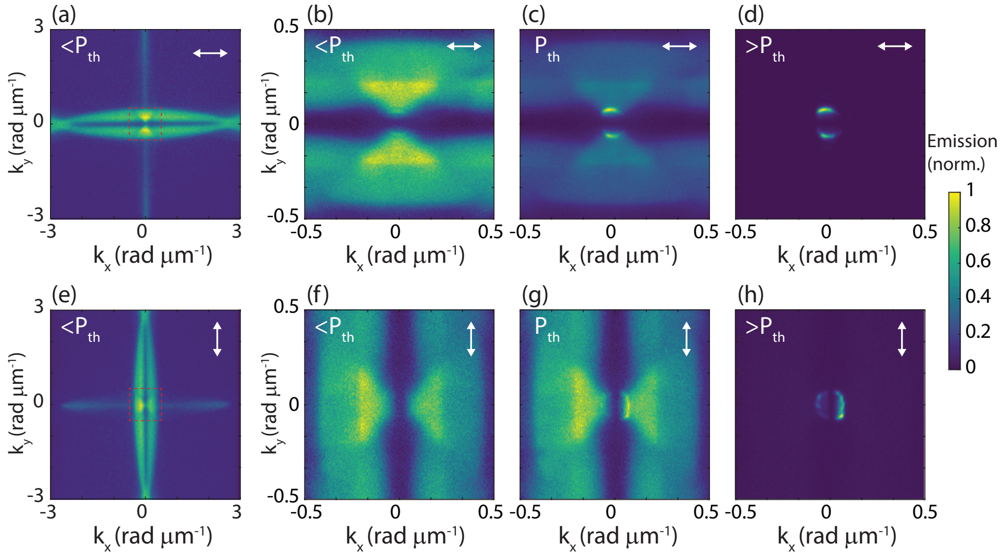

Figure S7: (a) x-polarized emission as a function of k_x and k_y below threshold. (b) Zoom of (a) around k=0 by using low NA objective. The distrubution of the emission changes on threshold (c) and above threshold (d). (e-g) same as (a-d) but when only collecting the y-polarized emission

References

[1] Andrey B. Evlyukhin, Carsten Reinhardt, Egor Evlyukhin, and Boris N. Chichkov. Multipole analysis of light scattering by arbitrary-shaped nanoparticles on a plane surface. *Journal of the Optical Society of America B*, 30(10):2589, 10 2013.

[2] Mengfei Wu, Son Tung Ha, Sushant Shendre, Emek G. Durmusoglu, Weon Kyu Koh, Diego R. Abujetas, José A. Sánchez-Gil, Ramón Paniagua-Domínguez, Hilmi Volkan Demir, and Arseniy I. Kuznetsov. Room-Temperature Lasing in Colloidal Nanoplatelets via Mie-Resonant Bound States in the Continuum. *Nano Letters*, 20(8):6005–6011, 8 2020.

[3] M G Moharam, Eric B Grann, Drew A Pommet, and T K Gaylord. Formulation for stable and efficient implementation of the rigorous coupled-wave analysis of binary gratings. *J. Opt. Soc. Am. A*, 12(5):1068–1076, 1995.

[4] Raymond Rumpf. *Design And Optimization Of Nano-optical Elements By Coupling Fabrication To Optical Behavior.* PhD thesis, University of Central Florida, 2006.

[5] Thomas Schuster, Johannes Ruoff, Norbert Kerwien, Stephan Rafler, and Wolfgang Osten. Normal vector method for convergence improvement using the RCWA for crossed gratings. *Journal of the Optical Society of America A*, 24(9):2880–2890, 2007.

[6] E. Palik. *Handbook of Optical Constants of Solids.* Academic Press, San Diego, CA, 1998.
